# The Tuberculosis Commission

**Published:** 1895-02

**Authors:** 


THE Tuberculosis Commission working under the direction of the State Board of Agriculture of New Jersey have just presented an official report of their work. The Commission have employed veterinarians in various parts of the State to carry out the provisions and rules of the commission. Tuberculin has been generally adopted as a diagnostic agent. The first herd tested was that of the State Hospital at Trenton. It was considered as a typically well-kept herd, numbering forty-eight in all; twenty-eight responded to the test, twenty-seven of which have been destroyed, and all but one presented marked evidence of the disease. Another herd of six cows, in a very emaciated state, gave generally the appearance physically of being afflicted with tuberculosis. Injections of tuberculin failed to give any reaction, and on slaughtering no evidence of the disease was visible. A number of other herds were tested and the reliability of the tuberculin test fully substantiated.

---

THE agitation for a better system of dealing with the contagious and infectious diseases of live stock of the State of Pennsylvania bids fair to end in the establishment of a department of agriculture, the secretary of which shall have a place in the Governor's cabinet, the departments of agriculture, dairy and food commission, forestry commission, zoology, weather bureau, and a State veterinarian, to be under the direction of this department.

